# E-cigarettes and non-suicidal self-injury: Prevalence of risk behavior and variation by substance inhaled

**DOI:** 10.3389/fpsyt.2022.911136

**Published:** 2022-09-06

**Authors:** Catherine W. Striley, Sara K. Nutley, Carolin C. Hoeflich

**Affiliations:** Department of Epidemiology, College of Public Health & Health Professions and College of Medicine, University of Florida, Gainesville, FL, United States

**Keywords:** non-suicidal and suicidal self-injurious thoughts and behaviors, e-cigarette, marijuana, conventional cigarette, substance use (drugs, vaping, alcohol)

## Abstract

**Background:**

Nicotine and cannabis inhalation through vaping or electronic delivery systems has surged among young adults in the United States, particularly during the coronavirus disease pandemic. Tobacco and marijuana use are associated with select adverse mental health outcomes, including symptoms of major depressive disorder and suicidal behaviors. Given the need for addiction specialists to treat problematic substance use with an integrated approach, the association between non-suicidal self-injury (NSSI) and use of e-cigarettes, tobacco, marijuana, and alcohol was examined among a diverse sample of college students.

**Methods:**

Healthy Minds Study data from 47,016 weighted observations, collected from college students in the 2018–2019 academic year, was used to explore associations between NSSI-related behaviors and past 30-day use of a vaping product (nicotine or marijuana). These relationships were assessed among those using vaping products only, and then among individuals using vaping products and alcohol, conventional cigarettes, and/or marijuana. Hierarchical logistic regression models estimating the relationship between vaping and NSSI were computed to adjust for the effects of demographic factors, symptomatology of psychiatric disorders, and concurrent use of other substances.

**Results:**

A fifth (22.9%) of respondents disclosed past 12-month NSSI; they were significantly more likely to screen positive for depression or anxiety compared to young adults without NSSI. Rates of using vaping products, conventional cigarettes, marijuana, or other substances were higher among students with NSSI even after controlling for potential cofounders. Additionally, students who used a THC-based liquid in their e-cigarettes were more likely to endorse NSSI in comparison to those who used “just flavoring.” However, young adults who vaped were less likely to disclose frequent NSSI-related behaviors than their peers who did not vape.

**Conclusions:**

These findings revealed an association between past 12-month NSSI and past 30-day vaping in a sample of young adults. Further surveillance among college populations and examination of potential sociodemographic confounders is necessary to confirm these findings and advance the substance use and addiction field.

## Introduction

Over the past 6 years, young adult use of e-cigarette and vaping products has increased to epidemic-level proportions (1–3). In part, this may be the consequence of vaping misinformation delivered via social media platforms and by peers who use e-cigarettes, outlets regarded by college-aged adults as credible sources of information about vaping products (4). In addition to positive social media messages promoting the use of e-cigarettes and other vaping products (5), multinational tobacco companies' marketing initiatives endorse e-cigarettes as a less harmful alternative to cigarette smoking (1, 6). However, research indicates both short- and long-term health risks associated with youth vaping (1, 2, 7–9), posing a particularly problematic public health concern.

E-cigarette use is associated with other risky health behaviors (e.g., binge drinking and use of other substances) and negative health outcomes (1, 2, 7, 9–17). Symptoms of mental health problems are also common among young people who use e-cigarettes. For instance, young adult use of vaping products has been associated with depression, disordered eating, ADHD, conduct disorder, anxiety, and PTSD (14, 18). Vaping nicotine alone or marijuana use alone or dual-use have all been associated with depressive symptoms and suicidal behaviors (19). Furthermore, a relationship has been observed between using e-cigarettes and suicidal ideation and attempts among youths. The use of e-cigarettes was associated with a 23% increased odds of seriously considering attempting suicide in the prior year among more than 25,000 adolescents participating in the US Youth Risk Behavior Survey (YRBS) (3). Dual-use increased the odds of suicidal behaviors even more in the same survey (3). Additionally, a medically serious suicide attempt was endorsed by one in 20 (5.6%) respondents endorsing e-cigarette use compared to fewer than one in 150 individuals who did not disclose e-cigarette use (0.6%) in a nationwide sample of Korean adolescents (20).

Non-suicidal self-injury (NSSI), defined as intentional and self-directed behavior(s) leading to physical harm without suicidal intent nor expectation of mortality (21–24), may also be linked to e-cigarette use/vaping. Approximately one in five (19.8%) college students in the United States (US) endorse past-12 month NSSI (25); pooled lifetime prevalence among adults between 18 and 24 years is slightly lower (13.4%) (26). While NSSI is highly heterogeneous in type, frequency, and severity (27), the behavior has been identified as a strong predictor of poor mental health outcomes, including stress, anxiety, and emotional dysregulation (28, 29). NSSI during adolescence increases the risk of attempting suicide during adulthood (30). Prior work has examined whether changes in the number of Google searches related to suicide is associated with changes in suicide rates (31). Importantly, NSSI during adolescence remains a significant risk factor for negative mental health in young adulthood, regardless of the frequency or stability of the behavior (29).

Despite emerging research reporting a positive association between vaping and marijuana use and suicidal behaviors, and specifically between vaping and suicide attempts, the relationship between substance use, NSSI, and suicidal behaviors remains understudied. This investigation aims to evaluate the relationship between the use of e-cigarettes, marijuana use, smoking, and alcohol use, and NSSI behavior using data collected from undergraduate and graduate students participating in the 2018–2019 Health Minds Study (HMS), an annual, internet-based survey assessing the mental health status and health care utilization of college students in North America. We hypothesized that students using e-cigarettes would be more likely to report NSSI behavior than those reporting no use and that the strength of this relationship would increase as the frequency of NSSI behavior increased. We further hypothesized that the strength of the relationship between e-cigarette use and NSSI would vary by the type of substance inhaled. Specifically, we postulated that students inhaling nicotine and marijuana-based e-liquids would be more likely to report NSSI, compared to those inhaling flavoring only. This study may assist clinicians in enhancing screening instruments to identify college populations at a greater risk for adverse mental health outcomes.

## Materials and methods

This secondary data analysis includes data collected from 35,777 undergraduate and graduate students participating in the HMS between Fall 2018 and Spring 2019. Since 2007, the HMS team has collected information from more than 400,000 students attending 350 colleges and universities primarily located in the United States. Participant recruitment and data collection methods have been described elsewhere (32, 33).

In short, random samples of 4,000–20,000 degree-seeking students at large, participating institutions (or all students at smaller institutions), 18 years of age and older, were recruited to participate via email invitations. Invitations provided a personalized link to a web page with more information on the study and an informed consent page. The page indicated the study purpose was to examine mental health and related issues as well as service utilization among college students. Between 2018 and 2019, approximately 16% of students who were invited to participate completed the web-based survey. To reduce non-response bias, the HMS team has constructed non-response weights using administrative data on full student populations (variables include gender, race/ethnicity, academic level, and grade point average). Accordingly, this data analysis includes 47,016 weighted observations (35,777 observations) that provided information related to substance use behavior, non-suicidal self-harm, and mental health symptomatology.

### Demographics

Students were asked to provide their age (coded as 18–25, 25+), gender (coded as male, female, trans male/trans man, trans female/transwoman, genderqueer/gender non-conforming, self-identify; recoded as male, female), and race (coded as African American/Black, American Indian or Alaskan Native, Asian American/Asian, Hispanic/Latino/a, Native Hawaiian or Pacific Islander, Middle Eastern/Arab/Arab American, White, Other; recoded as white, non-white). While the information from students who did not identify as male or female would be extremely valuable to the field, due to the small sample size in the Healthy Minds sample (trans male/trans man: *N* = 173, trans female/transwoman: *N* = 81, genderqueer/gender non-conforming: *N* = 500, self-identify: *N* = 382), only those who identified as male or female were included in this analysis.

### Non-suicidal self-injurious behaviors (NSSI)

Students were provided a list of non-suicidal self-injurious behaviors and were asked to consider ways they may have hurt themselves on purpose, without intending to kill themselves. Past 12-month history of the following behaviors was assessed: (1) cutting oneself, (2) burning oneself, (3) punching or banging oneself, (4) scratching oneself, (5) biting oneself, (6) pulling one's hair, (7) punching or banging an object, (8) interference with wound healing, (9) carving words or symbols into the skin, and (10) rubbing sharp objects into the skin. Participants were provided a comment field to specify other self-injurious behaviors as appropriate. Students who reported past 12-month NSSI were asked about the frequency of self-injurious behavior over the last year (once or twice, once a month or less, 2 or 3 times a month, once or twice a week, 3 to 5 days a week, nearly everyday or everyday). A two-category variable was created to denote frequent NSSI behavior (i.e., behavior occurring 2 or 3 times a month or more) or no frequent NSSI behavior (behavior occurring less than that).

### E-cigarette use

Students were asked about their past 30-day use of e-cigarettes and vaping products. Respondents who endorsed past 30-day use were classified as students who currently use an e-cigarette. These individuals were further queried about the type of mist inhaled at last use and were asked to select one of the following: nicotine, marijuana (hereafter referred to as THC for Delta-9-tetrahydrocannabinol), “just flavoring,” or any vaping. Those who did not endorse past 30-day use were classified as not currently using e-cigarettes.

### Use of other substances

To assess the use of conventional cigarettes, participants were asked to report the number of cigarettes smoked per day over the last 30 days. Students smoking one or more cigarettes per day were classified as currently using conventional cigarettes and those who reported smoking zero cigarettes were classified as individuals who did not use conventional cigarettes. Students were also asked about past 30-day use of marijuana (yes/no) and past 30-day use of other substances (yes/no), including cocaine, heroin, methamphetamines, ecstasy, non-prescribed opioid pain relievers (such as Vicodin and OxyContin), non-prescribed stimulants (such as Ritalin and Adderall), and other drugs without a prescription. Finally, students were asked to report past 2-week alcohol use (yes/no).

### Psychiatric symptomatology

Students completed the Patient Health Questionnaire (PHQ-9) (34, 35) and the Generalized Anxiety Disorder 7-item (GAD-7) scale (36) to assess the current symptomatology of depression and anxiety, respectively. Those with a PHQ-9 total score of 15 or greater were classified as having moderately severe or severe depression, and those with a GAD-7 total score ≥10 were classified as having moderate or severe anxiety. Scores below the cut points were classified as not having moderately severe depression or anxiety.

### Statistical analysis

Using Pearson's chi-squared tests, we compared the demographic characteristics, psychiatric symptomatology, and substance use behaviors of students endorsing past 12-month NSSI to that of students endorsing no self-injury (Tables 1, 2). Variation in substance use behavior was assessed among college students reporting NSSI and suicidal ideation, those reporting NSSI only (i.e., no suicidal ideation), and those reporting neither NSSI nor suicidal ideation (Table 3; Analysis limited to students who provided information regarding past-year suicidal intent [In the past year, did you ever seriously think about attempting suicide? (yes/no)], *N* = 46,883). Additionally, among those endorsing the use of e-cigarettes, we considered whether students endorsing both e-cigarette use and other substance use behavior were more likely to report NSSI than those using e-cigarettes alone (Table 4).

**Table 1:** Demographic overview of study sample, by past 12-month self-injury.

	**Past-year NSSI** ***N* = 10,757** **(%)**	**No past-year NSSI** ***N* = 36,259** **(%)**	***p***
**Gender**			<0.0001
Male	4,143 (38.5)	15,741 (43.4)	
Female	6,613 (61.5)	20,517 (56.6)	
**Age**			<0.0001
18–25 years	9,673 (89.9)	27,657 (76.3)	
26+ years	1,084 (10.1)	8,601 (23.7)	
**Race**			0.0084
White	7,027 (65.3)	22,793 (62.9)	
Non-white	3,730 (34.7)	13,466 (37.1)	
**Depression symptoms**			<0.0001
Moderate–Severe	4,198 (39.0)	4,164 (11.5)	
None–Mild	6,559 (61.0)	32,095 (88.5)	
**Anxiety symptoms**			<0.0001
Moderate–Severe	6,009 (55.9)	8,678 (23.9)	
None–Mild	4,748 (44.1)	27,581 (76.1)	

**Table 2:** Substance use behavior, by past 12-month self-injury.

	**Past-year** **self-injury** ***N* = 10,757** **(%)**	**No past-year** **self-injury** ***N* = 36,259** **(%)**	***p***
Electronic cigarette use	2,901 (27.0)	5,854 (16.1)	<0.0001
Conventional cigarette use	1,747 (16.2)	3,088 (8.5)	<0.0001
Marijuana use	4,048 (37.6)	7,156 (19.7)	<0.0001
Alcohol use	6,615 (61.5)	20,571 (56.7)	<0.0001
Use of other substances	966 (9.0)	1,428 (3.9)	<0.0001

**Table 3:** Substance use behavior, by past 12-month NSSI **AND suicidal ideation** (*N* = 46,883).

	**NSSI +** **Ideation** ***N* = 3,932** **(%)**	**NSSI Only** ***N* = 6,794** **(%)**	**No NSSI** ***N* = 36,157** **(%)**	***p***
E-cig use	1,158 (29.4)	1,726 (25.4)	5,847 (16.2)	<0.0001
Conv. Cig use	742 (18.9)	1,001 (14.7)	3,070 (8.5)	<0.0001
Marj. use	1,648 (41.9)	2,389 (35.2)	7,142 (19.8)	<0.0001
Alc. use	2,455 (62.4)	4,137 (60.9)	20,515 (56.7)	<0.0001
Other Sub. use	426 (10.8)	540 (7.9)	1,425 (3.9)	<0.0001

**Table 4:** Concurrent substance use behavior **among individuals using e-cigarettes (*N*** **=** **8,755)**, by past 12-month NSSI.

	**Past-year NSSI** ***N* = 2,901** **(%)**	**No past-year NSSI** ***N* = 5,854** **(%)**	***p***
Conventional cigarette use			<0.0001
E-cig – Conv. Cig use	1,795 (61.9)	4,433 (75.7)	
E-cig + Conv. Cig use	1,106 (38.1)	1,421 (24.3)	
Marijuana use			<0.0001
E-cig – Marj. Use	903 (31.3)	2,760 (47.2)	
E-cig + Marj. Cig	1,998 (68.9)	3,094 (52.8)	
Alcohol use			0.7242
E-cig – Alc. use	539 (18.6)	1,058 (18.1)	
E-cig + Alc. Use	2,361 (81.4)	4,796 (81.9)	
Use of other substances			<0.0001
E-cig – Other Sub.	2,280 (78.6)	5,078 (86.8)	
E-cig + Other Sub.	621 (21.4)	775 (13.2)	
Number of substances used			<0.0001
E-cig use only	198 (6.8)	613 (10.5)	
E-cig + 1–2 substances	1,746 (60.2)	4,131 (70.6)	
E-Cig + 3–4 substances	926 (31.9)	1,081 (18.5)	
E-Cig + 5+ substances	31 (1.1)	29 (0.5)	

Three sets of hierarchical logistic regression models were then used to assess the relationship between the use of e-cigarettes and past 12-month NSSI (Table 5). In the first set of models, the unadjusted association between past 30-day use of e-cigarettes and NSSI was assessed, and three additional models successively added the effects of (1) demographic characteristics, (2) psychiatric symptomatology, and (3) use of other substances. In the second set of models, we assessed whether the type of e-liquid inhaled predicted the odds of past 12-month NSSI among those using e-cigarettes, again controlling for the effects of demographic characteristics, psychiatric symptoms, and substance use behavior. In the final set of models, the association between the use of e-cigarettes and NSSI frequency was assessed among those reporting any past 12-month self-injurious behavior, adjusting for the aforementioned covariates.

**Table 5:** Hierarchical logistic regression models.

**E-cigarette use and past 12-month NSSI (weighted** ***N*** = **47,016)**				
	**Model 1** **OR (95% CI)**	**Model 2** **aOR (95% CI)**	**Model 3** **aOR (95% CI)**	**Model 4** **aOR (95% CI)**
**Exposure of interest**				
Electronic cigarette use	1.92 (1.76, 2.10)	1.77 (1.61, 1.93)	1.54 (1.40, 1.70)	1.27 (1.14, 1.40)
**Demographics**				
Gender (Female)	–	1.29 (1.19, 1.40)	1.07 (0.98, 1.16)	1.09 (0.99, 1.18)
Age (18–25 years)	–	2.58 (2.29, 2.91)	2.49 (2.20, 2.82)	2.60 (2.30, 2.95)
Race (White)	–	1.02 (0.94, 1.11)	–	–
**Psychiatric symptoms**				
Moderate–Severe depression	–	–	2.76 (2.48, 3.06)	2.69 (2.42, 2.99)
Moderate–Severe anxiety	–	–	2.46 (2.24, 2.70)	2.45 (2.32, 2.69)
**Substance use**				
Conventional cigarette use	–	–	–	1.67 (1.46, 1.90)
Alcohol use	–	–	–	1.07 (0.98, 1.16)
Use of other substances^*^	–	–	–	1.49 (1.26, 1.75)
**E-liquid inhaled and past 12-month NSSI (weighted** ***N*** **=** **8,358)**				
**Exposure of interest**				
THC E-Liquid (vs. “just flavoring”)	1.69 (1.19 2.40)	1.81 (1.27, 2.58)	1.82 (1.23, 2.70)	1.66 (1.11, 2.47)
Nicotine E-Liquid (vs. “just flavoring”)	1.23 (0.90, 1.68)	1.32 (0.96, 1.82)	1.36 (0.96, 1.93)	1.09 (0.77, 1.54)
**Demographics**				
Gender (Female)	–	1.38 (1.17, 1.64)	1.11 (0.92, 1.32)	–
Age (18–25 years)	–	2.34 (1.69, 3.24)	2.35 (1.63, 3.38)	2.53 (1.73, 3.70)
Race (White)	–	0.74 (0.62, 0.88)	0.83 (0.68, 1.00)	–
**Psychiatric symptoms**				
Moderate–Severe depression	–	–	2.80 (2.27, 3.45)	2.73 (2.21, 3.36)
Moderate–Severe anxiety	–	–	2.36 (1.94, 2.87)	2.42 (1.99, 2.94)
**Substance use**				
Conventional cigarette use	–	–	–	1.87 (1.53, 2.27)
Alcohol use	–	–	–	0.92 (0.73, 1.15)
Use of other substances^*^	–	–	–	1.41 (1.12, 1.78)
**E-cigarette use and frequent NSSI (i.e., 2 or more times per month; weighted** ***N*** **=** **10,354)**				
**Exposure of interest**				
Electronic cigarette use	0.77 (0.63, 0.93)	0.76 (0.62, 0.92)	0.69 (0.57, 0.83)	0.73 (0.60, 0.90)
**Demographics**				
Gender (Female)	–	1.00 (0.83, 1.20)	–	–
Age (18–25 years)	–	1.25 (0.94, 1.70)	–	–
Race (White)	–	1.02 (0.85, 1.21)	–	–
**Psychiatric symptoms**				
Moderate–Severe depression	–	–	2.06 (1.68, 2.53)	2.05 (1.67, 2.52)
Moderate–Severe anxiety	–	–	1.52 (1.23, 1.88)	1.51 (1.22, 1.88)
**Substance use**				
Conventional cigarette use	–	–	–	0.95 (0.75, 1.22)
Alcohol use	–	–	–	0.77 (0.65, 0.92)
Use of other substances^*^	–	–	–	1.10 (0.81, 1.49)

* Not including marijuana.

## Results

One in five students (*N* = 10,756.8 [rounded to 10,757], 22.9%) endorsed past 12-month non-suicidal self-injury, of whom 21.3% (*N* = 2,296) reported NSSI behavior 2 or more times per month. Compared to those who reported no self-injurious behavior (*N* = 36,258.5 [rounded to 36,259]), students reporting past 12-month NSSI were more likely to be female (61.5% vs. 56.6%, *p* < 0.0001), White (65.3% vs. 62.9%, *p* < 0.0001), and between the ages of 18 and 25 (89.9% vs. 76.3%, *p* < 0.0001; Table 1). Those reporting NSSI were also more than twice as likely to report moderate or severe anxiety symptoms (55.9% vs. 23.9%, *p* < 0.0001) and over three times as likely to report moderately severe or severe symptoms of depression (39.0% vs. 11.5%, *p* < 0.0001) when compared to peers who did not endorse NSSI.

### Substance use

In comparison to those who reported no self-injurious behavior, participants reporting NSSI more frequently endorsed the use of all substances, including electronic cigarettes (Table 2). In particular, more than one in four students reporting NSSI behavior also reported the use of e-cigarettes, compared to one in six students who did not report NSSI. Further, those endorsing past 12-month NSSI were approximately twice as likely to report the use of conventional cigarettes (16.2% vs. 8.5%, *p* < 0.0001) and marijuana (37.6 vs. 19.7%, *p* < 0.0001), and nearly three times as likely to report the use of other substances (9.0% vs. 3.9%, *p* < 0.0001). Students endorsing NSSI were also slightly more likely to report past 2-week alcohol consumption (61.5% vs. 56.7%, *p* < 0.0001) compared to those reporting no self-injurious behavior.

When compared to students who endorsed NSSI only (no suicidal ideation) and those with neither NSSI nor suicidal ideation, participants who reported both past 12-month NSSI and suicidal ideation were more likely to report use of e-cigarettes (NSSI + suicidal ideation: 29.4%; NSSI only: 25.4%; no NSSI: 16.2%; *p* < 0.0001), conventional cigarettes (NSSI + suicidal ideation: 18.9%; NSSI only: 14.7%; no NSSI: 8.5%; *p* < 0.0001), marijuana (NSSI + suicidal ideation: 41.9%; NSSI only: 35.2%; no NSSI: 19.8%; *p* < 0.0001), alcohol (NSSI + suicidal ideation: 62.4%; NSSI only: 60.9%; no NSSI: 56.7%; *p* < 0.0001), and other substances (NSSI + suicidal ideation: 10.8%; NSSI only: 7.9%; no NSSI: 3.6%; *p* < 0.0001) (Table 3).

### Multiple substance use

To further understand the relationship between substance use behaviors, including vaping, and NSSI, we assessed variation in current substance use behavior by NSSI among those who endorsed vaping (*N* = 8,755). Specifically, we assessed the relationships between the use of conventional cigarettes only, marijuana only, alcohol only, and other substance (none, 1–2, 3–4, 5+) and NSSI among students reporting the use of e-cigarettes. In comparison to those who reported no self-injurious behavior, participants who endorsed NSSI were significantly more likely to report the use of both e-cigarettes and conventional cigarettes (38.1% vs. 24.3%, *p* < 0.0001), as well as the use of both e-cigarettes and marijuana (68.9% vs. 52.8%, *p* < 0.0001). However, those reporting NSSI were no more likely to report the use of both e-cigarettes and alcohol than those who reported no NSSI behavior. When the number of additional substances used was summed, those endorsing NSSI were almost twice as likely as those without NSSI to report the use of e-cigarettes and 3–4 or 5+ other substances (31.9% vs. 18.5% and 1.1% vs. 0.5% respectively, both *p* < 0.0001; Table 4).

### Hierarchical logistic regression models

The results of all hierarchical logistic regression models are displayed in Table 5. In our first set of hierarchical logistic regression models assessing the association between past 30-day use of e-cigarettes and NSSI, we found that past 30-day e-cigarette use increased the odds of NSSI 2-fold (OR: 1.92, 95% CI: [1.76, 2.10], *p* < 0.0001). The size of the effect was reduced incrementally with adjustment for (1) demographic characteristics (aOR: 1.77, 95% CI: [1.61, 1.93], *p* < 0.0001), (2) psychiatric symptomatology (aOR: 1.54, 95% CI: [1.40, 1.70], *p* < 0.0001), and (3) co-occurring substance use behavior (aOR: 1.27, 95% CI: [1.14, 1.40], *p* < 0.0001). However, the relationship between e-cigarette use and NSSI remained significant in all models.

In our second set of hierarchical logistic regression models assessing the relationship between the type of e-liquid inhaled and NSSI among individuals using e-cigarette(s), we found that individuals vaping THC-based e-liquids were 66% more likely to report past 12-month NSSI than students vaping “just flavoring” after controlling for relevant covariates (aOR: 1.66, 95% CI: [1.11, 2.47], *p* = 0.0127). However, we observed no difference in NSSI behavior between students vaping nicotine-based e-liquids and those vaping “just flavoring” (aOR: 1.09, 95% CI: [0.77, 1.54], *p* = 0.6359).

In our final set of hierarchical logistic regression models evaluating the association between use of e-cigarettes and NSSI frequency among those reporting any past 12-month self-injurious behavior, students using electronic cigarettes were less likely to report frequent self-injurious behavior (i.e., 2 or more times per month) compared to those not using e-cigarettes (OR: 0.77, 95% CI: [0.63, 0.93], *p* = 0.0058). This relationship remained significant after controlling for (1) demographic characteristics (aOR: 0.76, 95% CI: [0.62, 0.92], *p* = 0.0045), (2) psychiatric symptomatology (aOR: 0.69, 95% CI: [0.57, 0.83], *p* = 0.0001), and (3) co-occurring substance use behavior (aOR: 0.73, 95% CI: [0.60, 0.90], *p* = 0.0023). Of note, conventional cigarette use and use of other substances were not significant predictors of NSSI frequency.

### Types of NSSI behavior endorsed by students using e-cigarette(s)

Pearson's chi-squared tests were used to examine the types of NSSI behaviors most frequently endorsed by students using e-cigarettes and those not using e-cigarettes. For almost all behaviors assessed, those using e-cigarettes were nearly twice as likely to endorse NSSI compared to individuals not using e-cigarettes. Among both students using e-cigarettes and those not using e-cigarettes, the most frequently endorsed self-injurious behaviors included (1) punching or banging oneself (13.4% vs. 7.6%, *p* < 0.0001), (2) punching or banging an object to hurt oneself (12.6% vs. 6.0%, *p* < 0.0001), (3) scratching oneself (12.0% vs. 8.2%, *p* < 0.0001), (4) pulling one's own hair (11.0% vs. 6.8%, *p* < 0.0001), (5) interference with wound healing (10.6% vs. 6.1%, *p* < 0.0001), and (6) cutting oneself (10.1% vs. 4.6%, *p* < 0.0001).

## Discussion

Using a large, national sample of college students, we examined associations between specific types of deliberate self-injurious behaviors and use of e-cigarettes, including specific types of liquid used in these devices, conventional cigarettes, and alcohol use. With almost 11,000 students endorsing deliberate self-injurious behavior in the prior year, we also had the power to comprehensively characterize the demographic and mental health profile of students who engage in NSSI and to evaluate the frequency of other substance use behaviors in this population. All variables were significantly different between those with past-year NSSI and with no endorsed NSSI, with younger, White women who endorsed moderate to severe symptoms of anxiety and depression more likely to report self-injurious behavior. When assessed individually, past-year NSSI was also more likely to be disclosed by those who also endorsed the use of e-cigarettes, conventional cigarettes, marijuana, alcohol, or other substances.

None of these results are surprising; rather they confirm what is already known among other samples. Prior literature indicates much higher rates of NSSI among youths using conventional cigarettes, THC, and other illicit substances (37). Indeed, in a systematic review of 36 studies investigating the relationship between substance use and self-injurious behavior in non-clinical samples, all but four studies found substance use to be significantly associated with self-injury (38). Some have postulated a link between substance use and NSSI through emotional (39) or affective (38) dysregulation, although the present study is not able to elucidate the mechanisms underlying the observed relationships. Nevertheless, the association between NSSI and e-cigarette use is concerning and suggests that addiction specialists should screen young adults using e-cigarette(s) for self-injurious behaviors. Combined with prior literature, our findings also support the need for integrative addiction medicine teams to ensure that patients, particularly college students, who would benefit from both mental health and substance use treatment, receive timely and adequate care.

We added to the literature by clarifying the relationship between e-cigarette use and other drug use and NSSI, and then by developing a set of hierarchical logistic regression models that display the continued importance of the association between vaping and past 12-month NSSI when controlling for all the other significant variables. Importantly, alcohol use did not add to the risk of NSSI while all other additional substances did, both independently and when added together. Indeed, using 3 or more substances in addition to e-cigarette use nearly doubled the risk of NSSI. Hierarchical models showed that risk for NSSI was heightened among younger, White women with affective symptoms, but that beyond these characteristics, engaging in vaping, smoking, and other substance use each contributed uniquely to predict NSSI.

Further, because of the increased recognition that young adults commonly vape liquid substances such as THC and nicotine, we explored whether e-cigarette users' risk for past 12-month NSSI varied by type of substance inhaled: nicotine, THC, or e-liquid believed to be substance-free (i.e., “just flavoring”). Importantly, vaping THC, but not nicotine, increased the risk for NSSI relative to vaping e-liquids containing flavoring only, again while controlling for demographic characteristics, affective symptoms, and other substances used. In this fully adjusted model, younger age, depression, and anxiety symptoms increased the risk for NSSI more than 2-fold to nearly 3 times the risk, though vaping THC and the use of conventional tobacco products or other substances continued to contribute to risk for self-injurious behavior. Clinicians treating college populations should consider screening students not only for e-cigarette use but also, for the specific types of liquids utilized in their vaping device.

Finally, we predicted frequent NSSI, which we defined as self-injurious behavior occurring 2 or more times each month over the past year. Contrary to our hypothesis, e-cigarette use decreased the risk for frequent NSSI in all four models. Interestingly, other substance use behavior did not affect the risk for frequent NSSI, though depression and anxiety increased the odds of frequent self-injurious behavior as expected. Although the temporal relationship between NSSI and substance use was unable to be assessed in the current study, a prior investigation of first-year college and university students suggested that the onset of a substance use disorder occurred more frequently after the onset of NSSI, compared to before NSSI onset (28). Thus, additional research on the motivations for e-cigarette use and the timing of use in relation to NSSI is necessary to advance the clinical relevance of future work in this field.

It is clear that young adult college students who are female, White, experience heightened symptoms of depression or anxiety; those who also engage in vaping, smoking, and other drug use are at increased risk for non-suicidal self-injurious behavior. Given our large sample size, we were also able to distinguish between nicotine and THC vaping and to individually calculate increased risk by type of liquid vaped. Uniquely, we found the risk increased among those who vaped THC, not nicotine, compared to flavor alone. This type of analysis is rare and will need to be replicated in future investigations given that most studies have not distinguished the type of liquid vaped when considering e-cigarette use and a risk factor for other health behaviors.

Despite its clinical salience, several limitations were present in this study. First, this work only assessed young adults who attended university or college in selected, participating U.S. academic institutions; this limits the generalizability of these findings and raises the potential of selection bias Second, the response rate was low; however, it is typical of online surveys all of which are subject to potential non-response bias. Non-response weights were applied to account for known characteristics, but other differences might not have been captured. Third, there may also be unaccounted psychosocial factors that confound the observed association between vaping and NSSI. Fourth, we were limited by the answer formats in vaping, smoking, and other substance use patterns. It would have been preferable to be able to distinguish those who smoked cigarettes every day, for instance, from those who smoked once a month. We were only able to distinguish those who reported smoking from those who reported no smoking. Fifth, these analyses did not explore associations between NSSI and different e-cigarette use patterns, including the dose, frequency, and mixing of e-cigarette flavors. Finally, non-suicidal self-injurious behaviors were not measured via a validated and reliable questionnaire that focuses on NSSI-related behaviors. Nevertheless, the Healthy Minds Study provides one of the largest, most representative surveys of college student mental health and behavior.

Future work is necessary to validate the association between e-cigarette use and NSSI behavior, to further examine other influences on this association, and to quantify the influence of dosage, frequency, and specific substance vaped. Indeed, it will be important to distinguish the type of liquid vaped and the frequency of self-harming behavior to maximize the utility of prevention programs and interventions among youth. Overall, our findings expanded scientific knowledge on the relationship between increasingly common substance use behaviors, such as e-cigarette use, NSSI, and suicidal ideation; this may aid in the identification of individuals who may benefit from tailored services and resources. As such, this research may help inform intervention and harm reduction efforts.

## Data availability statement

Publicly available datasets were analyzed in this study. This data can be found here: Healthy Minds https://healthymindsnetwork.org/research/data-for-researchers/.

## Ethics statement

The University of Michigan's Health Sciences and Behavioral Sciences IRB approved the Healthy Minds study. All participants gave informed consent. Secondary analysis based on the publicly available data was approved by the University of Florida Institutional Review Board.

## Author contributions

CS conceptualized the study, guided the analysis and wrote the first draft of the discussion, edited and rewrote other sections, and approved the final manuscript. SN assisted with the conceptualization of the study, conducted the analysis, wrote the first draft of the Introduction, Methods, and Results, and revised the final manuscript. CH made substantial revisions and edits to the manuscript. All authors approved the final version of this paper.

## Funding

CH was supported by the UF Substance Abuse Training Center in Public Health from the National Institute of Drug Abuse (NIDA) of the National Institutes of Health under award number T32DA035167 (PI: Dr. Linda B. Cottler).

## Conflict of interest

The authors declare that the research was conducted in the absence of any commercial or financial relationships that could be construed as a potential conflict of interest.

## Publisher's note

All claims expressed in this article are solely those of the authors and do not necessarily represent those of their affiliated organizations, or those of the publisher, the editors and the reviewers. Any product that may be evaluated in this article, or claim that may be made by its manufacturer, is not guaranteed or endorsed by the publisher.

## Author disclaimer

The content is solely the responsibility of the authors and does not necessarily represent the official views of the National Institutes of Health.

## Nomenclature

NSSI: Non-suicidal self-injury;

THC: Tetrahydrocannabinol.
